# Is human cytomegalovirus associated with breast cancer progression?

**DOI:** 10.1186/1750-9378-8-12

**Published:** 2013-04-04

**Authors:** Dolores Utrera-Barillas, Hilda-Alicia Valdez-Salazar, David Gómez-Rangel, Isabel Alvarado-Cabrero, Penélope Aguilera, Alejandro Gómez-Delgado, Martha-Eugenia Ruiz-Tachiquin

**Affiliations:** 1 Medical Research Unit in Oncological Diseases, Oncology Hospital, XXI Century National Medical Center, Mexican Social Security Institute, Mexico City, 06720, Mexico; 2 Medical Research Unit in Infectious Diseases, Pediatrics Hospital, XXI Century National Medical Center, Mexican Social Security Institute, Mexico City, 06720, Mexico; 3 Department of Medical Oncology, Oncology Hospital, XXI Century National Medical Center, Mexican Social Security Institute, Mexico City, 06720, Mexico; 4 Department of Pathology, Oncology Hospital, XXI Century National Medical Center, Mexican Social Security Institute, Mexico City, 06720, Mexico; 5 Cerebrovascular Pathology Laboratory, National Institute of Neurology and Neurosurgery, Mexico City, 14269, Mexico; 6 Medical Research Unit in Human Genetics, Pediatrics Hospital, XXI Century National Medical Center, Mexican Social Security Institute, Mexico City, 06720, Mexico

**Keywords:** Breast, Cancer, Progression, Virus, Human cytomegalovirus, Polymerase chain reaction, DNA

## Abstract

**Background:**

It has been hypothesized that human cytomegalovirus (HCMV) may be associated with breast cancer progression. However, the role of HCMV infection in breast cancer remains controversial. We aimed to assess whether HCMV genes (*UL122* and *UL83*) could be detected in breast carcinomas and reinvestigated their possible association with breast cancer progression. DNA from paraffin-embedded tissues was analyzed by real-time PCR. We investigated 20 fibroadenomas and 27 primary breast carcinomas (stages II, III, and IV).

**Findings:**

Two carcinomas were positive for HCMV, one was positive for two TaqMan viral detection probes, and one was positive for a sole TaqMan viral detection probe (UL83), whereas the remainder of the samples was negative.

**Conclusions:**

Samples studied showed no association between HCMV infection and breast cancer progression.

## Introduction

The number of new cancer cases and deaths is expected to increase worldwide. In Mexico, breast cancer has an incidence of 21.2%, mortality of 13.2%, and 5-year prevalence of 30.8% [1]. Cases of breast cancer were recognized from the second decade of life and there was a peak incidence between 40 and 54 years of age. The majority of cases were at advanced stages (II, III, and IV). Metastasis is frequently a final and fatal step in the progression of solid malignancies. The molecular requirements for some of these steps may be specific tissues. Viruses have been central to modern cancer research and provide profound insight into both infectious and non-infectious cancer causes and players in disease progression. This diverse group of viruses reveals unexpected connections among innate immunity, immune sensors, and tumor suppressor signaling that control both viral infection and cancer. Infectious agents can be promoters of neoplastic transformation. Viruses associated with cancer (EBV, KSHV, HPV, HBV, HCV, HTLV-1, SV40, JCV, BKV, and MCV, HIV indirect carcinogen, HCMV oncomodulator, and controversial HMTV/MMTV) have been found to cause 16.1% of human cancers worldwide [2]. Human cytomegalovirus (HCMV) is a widespread opportunistic herpesvirus that causes severe and fatal diseases in immunocompromised individuals including those who are organ transplant recipients, HIV-infected and patients with cancer. *In vitro*, HCMV can transform cells and deregulate other pathways relevant to adenocarcinoma pathogenesis, especially those affecting the cell cycle, mutagenesis, apoptosis, and angiogenesis when it has an oncomodulator role [3]. The purpose of this study was to determine and investigate a possible association between progression of breast cancer and HCMV infection in our tumor collective of advanced breast carcinomas consisting of 27 primary breast carcinomas and 20 fibroadenomas by real-time PCR in order to compare malignant vs. non-malignant tumors.

## Materials and methods

Samples of archived paraffin-embedded breast tissue (years: 2001−2008) were obtained from the Oncology Hospital of the XXI Century National Medical Center, Mexican Social Security Institute. The local ethics and scientific research committees approved the protocol.

The study group was comprised of 27 patients with breast carcinomas (stages II, III, and IV) who had recurrent disease and progression to anthracyclines and taxanes. All samples had a minimum of 70% cancer cells. The control group was comprised of 20 samples of fibroadenomas.

### Genomic DNA extraction

To purify DNA from paraffin-embedded tissue, 10-μm-thick slices of carcinomas and fibroadenomas were subsequently treated with 900 μL of xylene to remove residual xylene. The tissue was washed twice with 1000 μL of 100% ethanol and was finally resuspended in 540 μL of RLT buffer (Qiagen). Dewaxed tissues were incubated three times with 60 μL of proteinase K at 56°C/24 h. DNA was extracted with AllPrep DNA/RNA/Protein Mini Kit (Qiagen) according to the manufacturer’s instructions. Briefly, lysated tissues were placed in a column and spun for 1 min/10000 rpm; later, the column was washed twice with AW1 and AW2 buffers. Finally, the DNA was eluted with 25 μL of EB buffer preheated to 70°C. DNA quantification was performed at 260 nm in Nanodrop (Thermo Scientific).

### Multiplex PCR for glyceraldehyde-3-phosphate-dehydrogenase

DNA integrity was established by the Multiplex PCR kit (Qiagen) for glyceraldehyde-3-phosphate-dehydrogenase (GADPH) amplification with increase of 100 bp (range: 100–700 bp) [4]. PCR was performed with 5 ng genomic DNA in a 12.5 μL volume containing 1X Master Mix and 0.2 μM of each primer (Table 1). Amplification was performed in a Biometra Thermocycler (T Professional) as follows: 95°C/15 min for 40 cycles at 94°C/30 sec; at 57°C/90 sec; at 72°C/90 sec and, finally, at 72°C/10 min. PCR products were separated by electrophoresis on agarose gel 2.0%, stained with 1X Gel network (Biotium) and visualized under an ultraviolet light transilluminator and photodocumenter system (Syngene).

**Table 1:** Sequence primers (multiplex PCR for glyceraldehyde-3-phosphate-dehydrogenase amplification)

**Forward primers**	**Reverse primers**	**Base pairs**
GTT CCA ATA TGA TTC CAC CC	CTC CTG GAA GAT GGT GAT GG	100
AGG TGG AGC GAG GCT AGC	TTT TGC GGT GGA AAT GTC CT	200
AGG TGA GAC ATT CTT GCT GG	TCC ACT AAC CAG TCA GCG TC	300
ACA GTC CAT GCC ATC ACT GC	GCT TGA CAA AGT GGT CGT TG	400
AGC CCC TAA GGT CTT CAA GC	CAT GCC TGT AGC TGG GAC TA	500
GGC TCC CTT GGG TAT ATG GT	GGA GCC AGT CTT GGA TGA	600
CCC CAC ACA CAT GCA CTT AC	AAT GAA GGG GTC ATT GAT GG	700

### Detection of HCMV by real-time PCR

HCMV detection was performed by real-time PCR (qPCR), amplifying a 96-bp fragment, immediate early gene expression, *Ie2* (UL122, forward primer: GGCTCACCTCGTCAATCTTGA; reverse primer: AGAAGGTGCGCAATATCATGAAAGA; TaqMan probe: FAM-CCCCCTTCTGCACACCC) and 84 bp, late gene expression, *pp65* (UL83, forward primer: GGGACACAACACCGTAAAGC; reverse primer: GTGGAAGAGGACCTAACGATGAC; TaqMan probe: FAM-CCGCAACCCTTCATGC). qPCR was performed with 100 ng of genomic DNA at a final volume of 8 μL: 1X TaqMan Universal PCR Master Mix (Applied Biosystems), 0.54 μL UL122- or 0.54 μL UL83- TaqMan probe. Amplification was performed in an ABI 7500 thermal cycler as follows: 60°C/2 min and 95°C/10 min, and 40 cycles: 95°C/15 sec and 60°C/1 min, and 60°C/30 sec. Positive control was done with HCMV strain AD-169 (ATCC VR-583). Negative controls were DNA white blood cells from healthy individuals (blood donors) and reaction mix without DNA. All qPCR reactions were carried out in duplicate.

### Statistical analysis

We used Fisher’s exact test to compare the number cytomegalovirus-positive samples between fibroadenomas and breast carcinomas.

## Results

The study group included subjects with a mean age of 53.26 ± 11.99 years (range: 27−76 years). Mean overall survival was 44 months (range: 12−84 months). All had progression with active tumor sites affecting bone, soft tissue, liver, lung, and central nervous system. The control group had a mean age of 45 ± 9.27 years (range: 27−67 years). Females with fibroadenoma were younger than females with carcinoma.

Sample 19 was HCMV-positive with both TaqMan probes: one detected an immediate early gene (synonym of viral replication) and the other detected a late gene (presence of virus). The sample ´eight´ only tested positive for a late gene. The remaining samples were HCMV-negative.

Patients positive for HCMV were detected in undifferentiated infiltrating ductal carcinomas and Luminal A molecular subtype. A 54-year-old patient had 49 months of surveillance: T4, IIIC, ER+, PR+, Her2/Neu-negative, and undetermined p53, whereas another patient aged 68 years had 54 months of surveillance: T4, IIB, ER+, PR+, Her2/Neu-negative, p53-positive. Patient data are shown in Table 2. There was no association (*p* = 0.50) between the presence of HCMV and the clinicopathological parameters (survival, progression, tumoral activity sites, alive without disease, alive with stable disease, alive without disease progression, died of disease) analyzed. Molecular detection was qualitative and HCMV-positive samples had a high number (>35) of threshold of detection (Ct) cycles.

**Table 2:** Clinicopathological features of breast cancer patients included in the study

**n = 27**	**Cases (%)**
Histological type	
Infiltrating ductal carcinoma	23 (86)
Lobular carcinoma	2 (7)
Mixed	2 (7)
Age (years)	
20–40	6 (22)
41–69	13 (48)
>60	8 (30)
Tumor size	
T0-T2	7 (26)
T3	9 (33)
T4	11 (41)
ER status	
Positive	16 (59)
Negative	8 (30)
NA	3 (11)
PR status	
Positive	13 (48)
Negative	11 (41)
NA	3 (11)
Her2/neu status	
Positive	8 (30)
Negative	11 (40)
NA	8 (30

T0-T2 tumor size <5 cm of diameter; T3 tumor size >5 cm of diameter; T4 tumor any size with extension to skin or chest.

ER, estrogen receptor; PR, progesterone receptor; NA, data not available.

## Discussion

**Figure 1:**
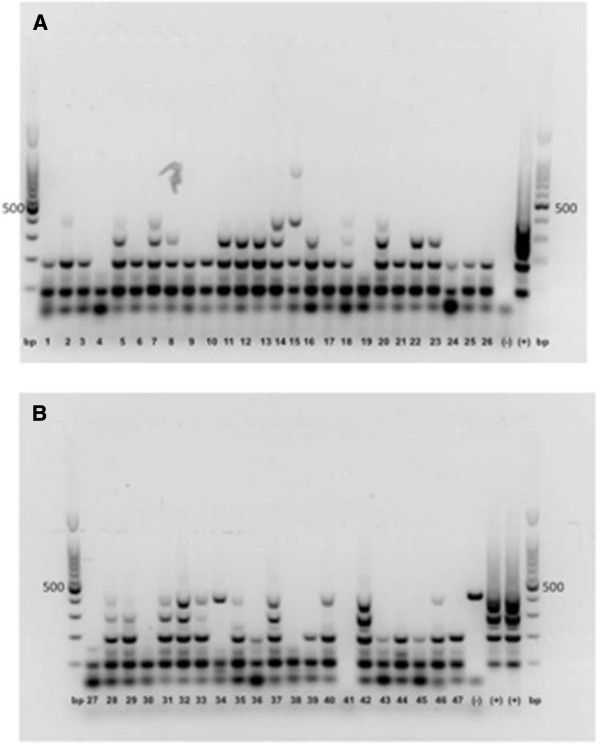
**Quality control for breast carcinomas and fibroadenomas.** Multiplex PCR for GADPH. (**A**) Lines 1−9 and 19−26 breast carcinomas; 10−18 fibroadenomas. (**B**) Lines 27, 37−45 breast carcinomas; 19−30, 46 and 47 fibroadenomas. Base pairs (bp); control positive (+), HCMV strain AD-169 (ATCC® VR-583); control negative (−), DNA white blood cells from healthy individual (blood donor) and reaction mix without DNA.

This study analyzed breast carcinoma ‘progression’ according to HCMV infection. By investigating progression of breast carcinoma resection specimens, we were able to study 27 primary tumors (stages II, III, and IV) and 20 fibroadenomas by qPCR. Several studies have been linked to HCMV infections with malignant phenotype, particularly with glioblastoma [5]. We are unable to support these latter findings with our collective tumors. Only two primary tumors (7.4%) from all 27 specimens (100%) were HCMV-positive qualitatively, with the remainder being negative. These results were coincident with colon cancer [6] but not coincident with other reports of breast cancer [7] where the target viral genes, molecular techniques and inclusion criteria of samples were different. The fact that we examined quality DNA from tumor specimens of breast carcinoma by Multiplex PCR for GAPDH [4] in all samples amplified a set of different-sized targets (Figure 1). PCR is able to detect acute as well as latent infections. PCR positivity in the two primary carcinomas of our tumor collective is due to replication and viral latency and the other to only a latent virus. Positivity was observed in the primary tumor. There was no positivity in the fibroadenomas. We were unable to support the finding that HCMV infection may be correlated with tumor progression. The only mechanism compatible with the observations reported is the “hit-and-run” hypothesis, which claims that a virus can mediate cellular transformation through an initial “hit”, whereas maintenance of the transformed state is compatible with viral molecule (“run”) loss. Although the concept of hit-and-run transformation has been controversial for many years, it remains the only plausible explanation for the observations of neoplastic transformation following *in vitro* transfection of herpesvirus and cytomegalovirus DNA, which have been presented by multiple laboratories for more than two decades [8].

## Conclusions

In the samples analyzed, there was no statistically significant association between malignant vs. non-malignant tumors (*p* = 0.50) and breast cancer progression vs. HCMV infection (*p* = 0.50), but we detected HCMV in two malignant tumors samples: one of which was positive for two detection probes, whereas the other sample was positive for one.

## Competing interests

The authors declare that they have no competing interests.

## Authors’ contributions

DUB conceived the study, coordinated and assisted in drafting the manuscript. HVS carried out molecular techniques and participated in the analysis of results. DGR was responsible for clinical aspects of the study and participated in the writing of the manuscript. IAC carried out the inclusion in paraffin of the tissues and immunohistochemistry assays for their classification. PA carried out molecular detection probes and helped to draft the manuscript. AGD performed the statistical analysis, critical review and editing of the manuscript. MERT conceived the study, participated in its design and coordination and was responsible for writing the manuscript and for acquiring financial support. All authors read and approved the final manuscript.
